# Beyond enzyme engineering: ordered enzyme immobilization drives enhanced productivity *in vitro*

**DOI:** 10.3389/fbioe.2026.1879695

**Published:** 2026-07-29

**Authors:** Matthew Wong, Thomas G. Neuman, Md Anarul Hoque, Sarah Moraïs, Edward Bayer, Georges Belfort, Mattheos Koffas

**Affiliations:** 1 Howard P. Isermann Department of Chemical and Biological Engineering and the Center for Biotechnology and Interdisciplinary Studies, Rensselaer Polytechnic Institute, Troy, NY, United States; 2 Faculty of Natural Sciences, Life Sciences, Ben-Gurion University of the Negev, Beer-Sheva, Israel; 3 Department of Biomolecular Sciences, The Weizmann Institute of Science, Rehovot, Israel

**Keywords:** biofuel synthesis, cell free, enhanced enzymatic productivity, immobilized enzymes, minimized diffusion limitations

## Abstract

Increasing environmental and economic pressures associated with global fossil fuel demand necessitate a shift toward sustainable fuel production. Production of second-generation biofuels, such as isobutanol, presents a promising opportunity; however, product toxicity limits *in vivo* production, motivating the development of optimized *in vitro* systems. As prior efforts have focused on enzyme engineering to improve titer, these systems remain constrained by diffusion-limited mass transfer. Here, we introduce an engineered, ordered cellulosome-based immobilization system that facilitates enhanced enzymatic productivity. Using keto-acid decarboxylase, alcohol dehydrogenase, and formate dehydrogenase as a model for the final steps of the isobutanol pathway, the system achieved a preliminary isobutanol titer of 5.92 g/L, a 78.4% yield, and an enzymatic productivity of 0.34 mL^-1^ h^-1^, representing marked improvement over previous cell-free approaches. This proof-of-concept approach introduces the importance of targeted immobilization alongside enzyme optimization and demonstrates ordered scaffoldin-mediated immobilization as a versatile platform approach for future *in vitro* biofuel and biochemical production.

## Graphical Abstract

**Figure:**
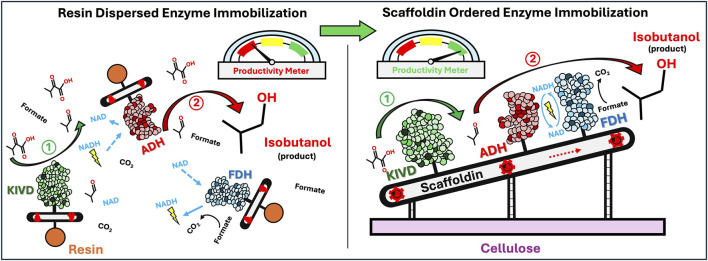

## 1 Introduction

Increasing global demand for fossil fuels has presented both environmental and economic challenges, including global warming and increasing fuel costs. Now more than ever, research and development of sustainable fuels are critical.

In response to this need, significant work has been done to examine the value of next-generation biofuels such as isobutanol (Dunlop, 2011; Chen and Liao, 2016; Su et al., 2020). However, a major limitation of *in vivo* isobutanol production is that alcohol is generally toxic to cellular systems. To address this challenge, previous work has explored the development of *in vitro* production systems that address this issue by utilizing immobilized enzymes on an epoxy resin (Grimaldi et al., 2014; Grimaldi et al., 2016). In this approach, a two-step enzymatic scheme was implemented: keto-acid decarboxylase (KIVD) catalyzed the conversion of ketoisovaleric acid (KIV) to isobutyraldehyde, which was then converted to isobutanol using alcohol dehydrogenase (ADH). NADH, the essential cofactor for the latter enzyme, was recycled by formate dehydrogenase (FDH).

Although this immobilized cell-free system demonstrated effective isobutanol production (2 g/L titer and 43% yield), the low overall yield necessitates improvement (Wong et al., 2019). We hypothesize that this limited performance resulted from the immobilization of pathway enzymes on separate resin particles, which imposed diffusion constraints on the transport of reaction intermediates. As a result, the reaction rate is governed by bulk-solution concentration, therefore limiting pathway efficiency.

A solution to the challenge imposed by epoxy immobilization is the utilization of a scaffoldin-based system, which results in specific and spatially organized enzymatic immobilization to enhance pathway efficiency (Liu et al., 2013; Lim et al., 2019). Here, this approach leverages the high affinity cohesin-dockerin interaction derived from cellulosome-producing bacteria (Moraïs et al., 2016a). In nature, this interaction is widely utilized by cellulosome-producing cellulolytic bacteria for the solubilization of cellulosic substrates (Bayer et al., 2004; Artzi et al., 2017). For isobutanol production, the enzymatic pathway is organized within a “scaffoldin” architecture consisting of multiple cohesin modules and a cellulose-binding module (CBM). Target enzymes are integrated within the architecture through genetic fusion with a docking module, known as a dockerin, which selectively assembles to the scaffoldin via specific cohesin-dockerin interactions (Bayer et al., 1994; Yaron et al., 1995).

**FIGURE 1:**
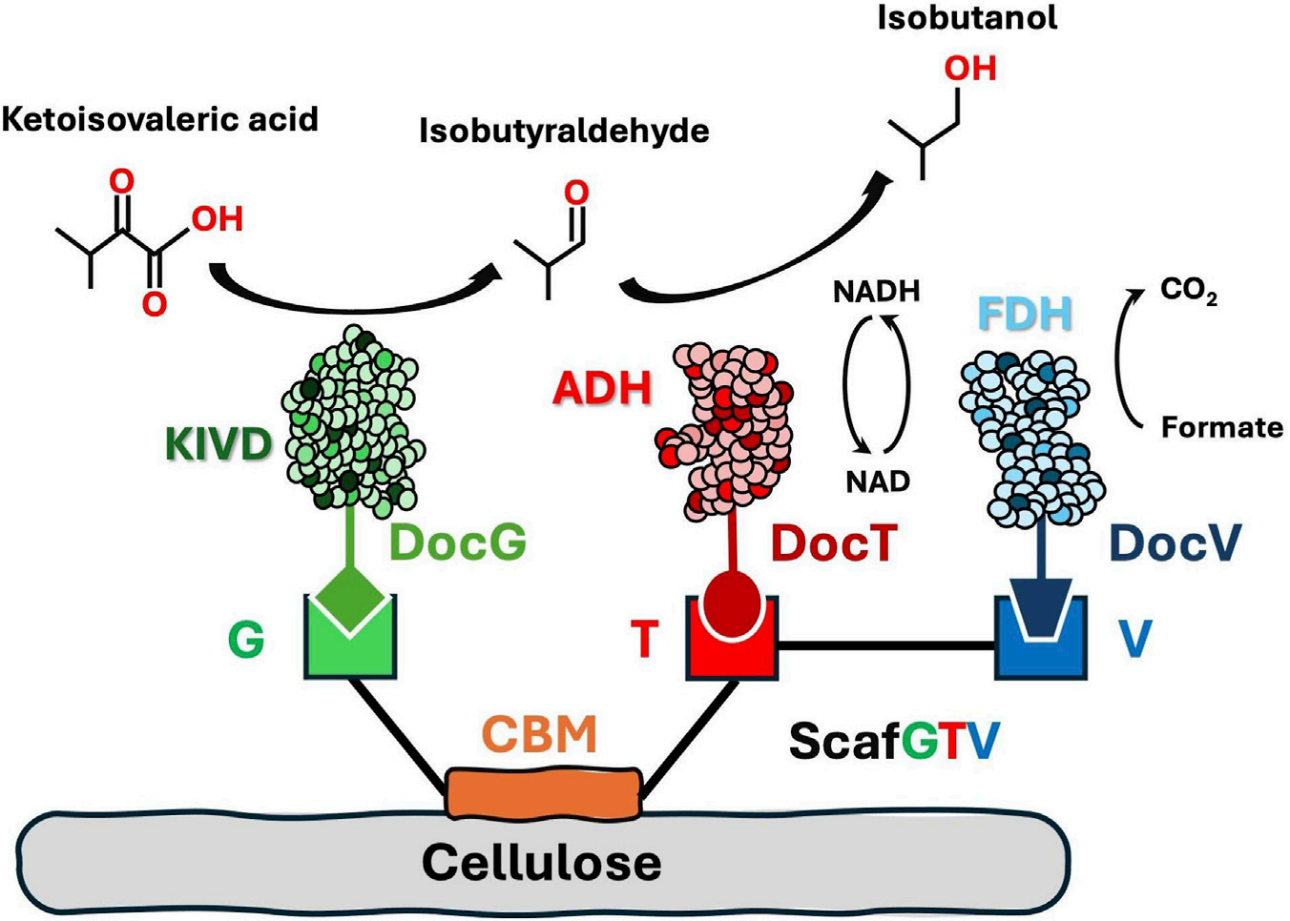
Schematic representation of cell-free production of isobutanol using the trivalent scaffoldin ScafGTV. For the *in vitro* production of isobutanol, the trivalent scaffoldin (ScafGTV), containing three cohesin domains (G, T, and V), is immobilized onto a cellulose support through its cellulose binding module (CBM). G, T, and V denote cohesins from *Archeoglobus fulgidus, Clostridium thermocellum,* and *Clostridium clariflavum,* respectively. Following immobilization, the pathway enzymes keto-acid decarboxylase (KIVD), alcohol dehydrogenase (ADH), and formate dehydrogenase (FDH) are attached to the scaffoldin through their corresponding dockerin/linker domains, DocG, DocT and DocV, respectively. Note, KIVD, ADH, and FDH are dimers but shown in monomer form for ease of visualization.

To develop a scaffoldin based enzymatic platform for the conversion of KIV into isobutanol (Figure 1), several enzymatic and expression system optimizations were performed. In terms of enzyme selection, a maltose-binding protein (MBP) derivative of the KIVD_LLM4_ variant was utilized to enhance solubility (via MBP) and stability (via the LLM4 variant of KIVD). Mutant ADH_29C8_ was selected over the wild type due to enhanced activity (Wong et al., 2019), while wild-type FDH was used based on its superior performance. Following enzyme selection, each enzyme in the pathway was genetically fused to a dockerin of distinct specificity, and their complementary cohesin-binding and catalytic activity features were examined experimentally. The dockerin-fused enzymes were then bound to corresponding cohesins of a trivalent (containing three cohesins) scaffoldin, which was itself bound to cellulose via the resident CBM. Immobilized cell-free production of isobutanol was then performed, resulting in a preliminary observation of a significant increase in both yield, titer, and productivity compared to the previously reported immobilized system (Wong et al., 2019).

## 2 Materials

Plasmids containing the genes for KIVD and mutants were provided by the Liao laboratory (Soh et al., 2017). Ketoisovaleric acid (KIV) was purchased from Sigma Aldrich (St. Louis, MO). Microcrystalline cellulose of 90 µm average particle size was provided by Asahi Kasei America (Ceolus™ UF-702, New York City, NY). pET9d containing a dockerin from *C. thermocellum* (DocT), pET28a containing a dockerin from *A. fulgidus* (DocG), pET28a containing a dockerin from *C. clariflavum* (DocV), and pET9d containing a trivalent scaffoldin (ScafGTV) with specificities that correspond to the aforementioned dockerins, were as described previously (Moraïs et al., 2016a). Dockerin linker gBlocks were purchased from IDT (Coralville, IA). NEBuilder was purchased from New England Biolabs (Ipswich, MA).

## 3 Methods

### 3.1 Fusion enzyme assembly

Each enzyme that was chosen for the pathway reaction was fused to a dockerin at either the enzyme’s N-terminus, initially utilizing a rigid linker (EAAAK)_3,_ or the C-terminus, utilizing the natural linker associated with each dockerin. The primers used for the cloning are listed in Supplementary Table S1. The N-terminal fusion of wild-type FDH (FDH_WT_) was further engineered as follows: adding a stop codon between the enzyme and its His-tag at the C-terminus; a His tag was added at the N-terminus of the fusion protein, and the (EAAAK)_3_ linker was replaced by either a short or long linker, as described previously (Caspi et al., 2009). The latter two variants were termed DocV-short-FDH_WT_ and DocV-Long-FDH_WT_, respectively. Cloning was done using the NEBuilder kit. Primers for this design are listed in Supplementary Table S2. Each plasmid was transformed into the *Escherichia coli* BL21(DE3) strain. The plasmid containing the C-terminal fusion for ADH was transformed into *E. coli* strains SoluBL21, pGro7, and pLysS to compare expression. Plasmids containing ADH and FDH fusions were transformed into pLysS for further experimentation.

### 3.2 Expression

Expression was conducted in the same manner as previously published for maltose binding protein (MBP) KIVD_LLM4_ fusions and the initial expression of ADH_29C8_ and FDH_WT_ fusions (Wong et al., 2019). Cell lines were cultured in 3 mL of Luria broth (LB) with 80 mg/mL of ampicillin overnight at 37 °C. These were transferred to 150 mL of fresh LB and shaken at 37 °C until an OD_600_ of 0.6-0.8 was reached. The cells were then induced with 0.5 mM isopropyl β-D-1-thiogalactopyranoside (IPTG) and then transferred to 30 °C for overnight incubation. Final expressions for fusions of ADH_29C8_ and FDH_WT_, as well as scaffoldin ScafGTV with KAN 50 mg/mL instead of AMP, were performed by shaking at 16 °C overnight (Moraïs et al., 2016b). SDS-PAGE was used to observe expression efficacy.

### 3.3 Purification

Purification of His-tagged fusion enzymes was conducted as published previously (Wong et al., 2019). Expressed cells were lysed via sonication at 70% amplitude, 10/5 on/off for 6 min and then centrifuged for 1 h at 10,000 g and 4 °C. The supernatant fluids were filtered and then deposited onto 2 mL nickel columns, which were shaken overnight at 4 °C. The resin was then washed with 50 mM imidazole in 20 mM Tris-HCl, 10 mM MgCl_2_, 100 mM NaCl at pH 7.4 and eluted with 200 mM imidazole in the same buffer. Enzymes were stored in a solution of 50% storage buffer (50 mM sodium phosphate, 5 mM MgCl_2_, 1.5 mM thiamine pyrophosphate, at pH 7.4) and 50% glycerol. ScafGTV was purified using a protocol adapted from Moraïs et al. (2016a). Cellulose was inserted into a gravity column and washed with six column volumes of Tris-buffered saline (TBS) (137 mM NaCl, 2.7 mM KCl, and 25 mM Tris-HCl, at pH 7.4) (Moraïs et al., 2016a). The scaffoldin-containing cells were lysed as described previously, with TBS instead of buffer A, and left shaking with the washed cellulose resin overnight at 4 °C. Afterwards, the flowthrough was collected, and the cellulose was washed with six volumes of TBS, followed by six column volumes of 1 M NaCl TBS, and finally, six column volumes of TBS again.

### 3.4 Activity assays

Activity assays were conducted in 96-well plates at ambient temperature (∼25 °C) with a total well volume for each assay of 200 µL. An aliquot (50 µL) of KIVD and FDH enzymes or 1 µL of ADH enzyme was first added to the well. Reagents were prepared in storage buffer. For KIVD enzymes, 86 mM KIV was utilized. For ADH enzymes, 200 µM of NADH was used as well as excess isobutanol. For FDH enzymes, 1.8 mM NAD was utilized as well as excess formate. Activity was measured over time using a microplate reader at 340 nm, the absorbance maxima for both KIV and NADH. Following absorbance measurement, activity was reported as the change in 340 nm absorbance, proportional to analyte concentration, over time relative to the enzyme mass.

### 3.5 Assessment of individual enzyme-scaffoldin binding

To determine the individual binding of the fusion enzymes to the cohesin-bearing scaffold, 500 mg (wet weight) of cellulose with bound ScafGTV was inserted into a 5 mL tube with 0.75 mL of the KIVD derivative of a stock enzyme mixture at a concentration of 0.51 mg/mL and incubated with shaking overnight at 4 °C. Similarly, 0.75 mL of the ADH or FDH derivatives (0.26 and 0.185 mg/mL, respectively) were added to separate tubes with bound ScafGTV for the binding assay. The post-incubation retentate, obtained by centrifuging the tube, was examined using SDS-PAGE to gauge if binding had occurred.

### 3.6 Pathway-scaffoldin immobilization

To confirm the immobilization of all three enzyme fusions to the cohesin-bearing scaffoldin, 500 mg (wet weight) of cellulose with bound ScafGTV was added to a 5 mL tube. The three dockerin-bearing enzymes, MBP-KIVDLLM4-DocG, ADH29C8-DocT, and DocV-Long-FDH^WT^, were mixed into the tube at the same concentration as those of the individual binding experiments (*i.e.,* 0.75 mL at 0.51, 0.26, and 0.185 mg/mL, respectively). The mixture of the three enzymes with the cellulose-bound ScafGTV was shaken overnight at 4 °C. The post-shaking retentate was examined using SDS-PAGE to determine if binding had occurred.

### 3.7 Production of isobutanol

Following binding of the enzymes to the immobilized scaffoldin as described above, the cellulose matrix was washed with storage buffer, shaken, centrifuged, and then decanted to remove excess unbound enzyme. A 3-mL mixture containing 102 mM KIV, 4 mM NADH, and a tenfold excess of formate in storage buffer was pipetted into the same 5 mL tube and then left shaking for 24 h at 37 °C. The tube was then centrifuged, and a pipette was used to remove the supernatant fluids, which were analyzed using HPLC as described previously (Gupta et al., 2022).

### 3.8 Productivity calculation

Process productivity is defined as the amount of product produced per unit reaction volume per unit time. Here, volumetric productivity, *P*_*v*_ (mg mL^-1^ h^-1^), is defined as:

$$P_{v}=\frac{C_{Isobutanol}}{t_{rxn}}$$ (1)

where $C_{Isobutanol}$ is the final titer of isobutanol (mg/mL) following a given reaction time [ $t_{rxn}$ (h)]. While this parameter provides an initial measure of process footprint of the metabolic pathway, it does not capture the dominant economic driver of *in vitro* reaction systems, enzymatic usage. Furthermore, volumetric productivity is insufficient for making system comparisons that differ in enzyme loading or reactor configuration.

To enable meaningful economic comparisons across volumetric scales, immobilization platforms, and reactor designs, productivity can be normalized by the total enzyme loading. Therefore, enzyme-normalized productivity, *P*_*e*_ (mL^-1^ h^-1^), defined as the volumetric productivity per unit enzyme mass, is given by:

$$P_{e}=\frac{C_{Isobutanol}}{m_{enzyme}\;t_{rxn}}$$ (2)

where *m*_*enzyme*_ (mg) is the total mass of enzyme used in the reaction.

## 4 Results and discussion

### 4.1 Expression of dockerin-containing fusion proteins

Unlike irreversible and generally random attachment approaches such as covalent binding to epoxy-resins, affinity interactions are reversible and highly selective. However, while traditional affinity approaches would promote specific binding of the enzyme to a target ligand, it is extremely challenging to induce site-specific ordering of a multiplicity of different enzymes on the same surface. The cohesin and dockerin system overcomes this limitation as a specific dockerin will predominantly bind to a particular cohesin counterpart within its own bacterial species (Pagès et al., 1997). These interactions exhibit high affinity (Slutzki et al., 2012) with dissociation constant (K_d_) values between 10^-8^ to 10^-11^ M, comparable to those of antibody-antigen interactions (Friguet et al., 1985). In addition, the modularity of the scaffoldin-based system provides flexibility in system design as alternative pathway enzymes can be fused to a specific dockerin and further immobilized.

To investigate the effect of dockerin placement on enzyme expression, each pathway enzyme was fused to a dockerin at either the N- or C-terminus. For C-terminal fusions, the natural linker found at the N-terminus of the dockerin gene was leveraged. In comparison, (EAAAK)_3_ was initially used for N-terminal fusions as it is a known linker that would keep domains separated and possibly increase stability (Chen et al., 2013).

**FIGURE 2:**
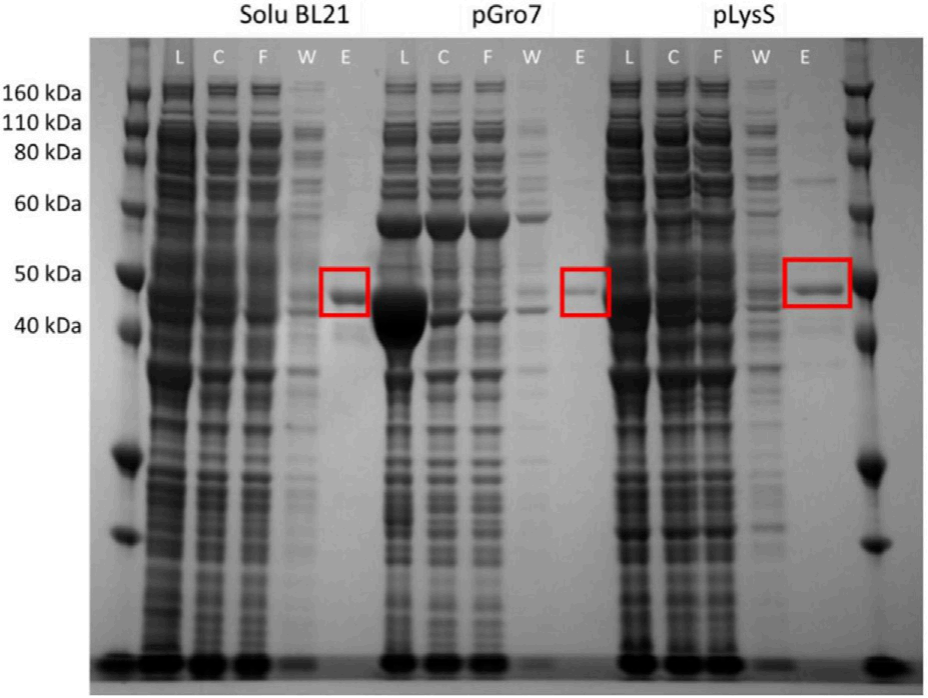
SDS-PAGE gel of ADH_29C8_-DocT derived from different cell lines. L: lysate, C: clarified lysate, F: flowthrough, W: wash, E: elution, where the lysate represents the sample taken directly from post sonication, clarified lysate follows the initial filtering step, and elution with 200 mM imidazole. The expressed bands are highlighted by the red boxes. The estimated MW of ADH_29C8_-DocT is 45 kDa. The cell lines used were SoluBL21, BL21 (DE3) pGro7, and BL21 (DE3) pLysS.

Initial expression of the N-terminal dockerin-containing fusion proteins using the *E. coli* BL21 strain was unsuccessful, except for the MBP-KIVD_LLM4_-DocG fusion protein (Supplementary Figure S1). Therefore, expression of the fused ADH and FDH enzymes was optimized in two ways. First, the expression temperature (Clark, 2001; Moraïs et al., 2016b) was reduced to slow enzyme synthesis and mitigate enzyme misfolding. Second, engineered *E. coli* strains that specialize in expressing recombinant proteins susceptible to folding or toxicity issues were selected, specifically, BL21 (DE3) pLysS, BL21 (DE3) pGro7, and SoluBL21. pLysS is a plasmid that expresses T7 lysozyme, which is known to inhibit basal expression, enabling better expression of proteins that utilize T7 RNA polymerase (Francis and Page, 2010). pGro7 is a plasmid that expresses additional chaperones, which aid in folding and help prevent protein aggregation (Callejon et al., 2017). SoluBL21 is an engineered BL21 strain, designed for expression of proteins with poor solubility (Weick et al., 2020). As a test case for enzyme expression, all 3 cell lines were confirmed to express the ADH-dockerin C-terminal fusion, ADH_29C8_-DocT (Figure 2).

**FIGURE 3:**
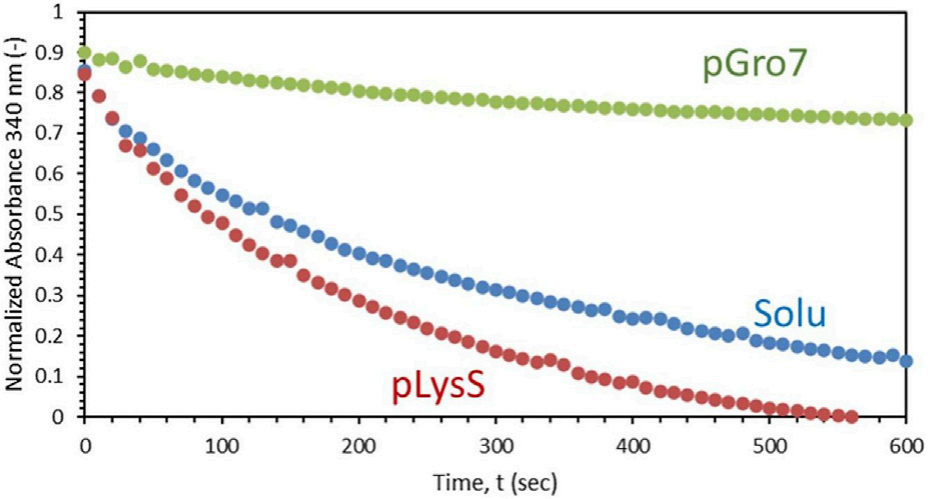
ADH_29C8_-DocT activity assay using protein expressed in three *E. coli* cell lines, i.e., SoluBL21, BL21 (DE3) pGro7, and BL21 (DE3) pLysS. Absorbance was normalized by dividing each value by the initial NADH absorbance.

Therefore, to identify the best expression system, enzymatic activity assays were conducted for ADH_29C8_-DocT expressed in the different strains (Figure 3). ADH_29C8_-DocT produced by pGro7 (5 abs*sec^-1^*mg^-1^), SoluBL21 (8.2 abs*sec^-1^*mg^-1^), and pLysS (5.5 abs*sec^-1^*mg^-1^) was active. Despite slightly lower catalytic activity, pLysS-expressed ADH_29C8_-DocT had measurably higher expression yield in comparison to the other two strains. Therefore, the BL21 (DE3) pLysS system was chosen for further low-temperature expressions. Using the BL21 (DE3) pLysS expression system, further expression of N-terminal DocT-ADH (Supplementary Figure S2) and FDH_WT_ fusions, containing a “short” or “long” linker (see Section 4.2), was also confirmed (Supplementary Figure S3).

### 4.2 Enzymatic activities of the fusion proteins

**FIGURE 4:**
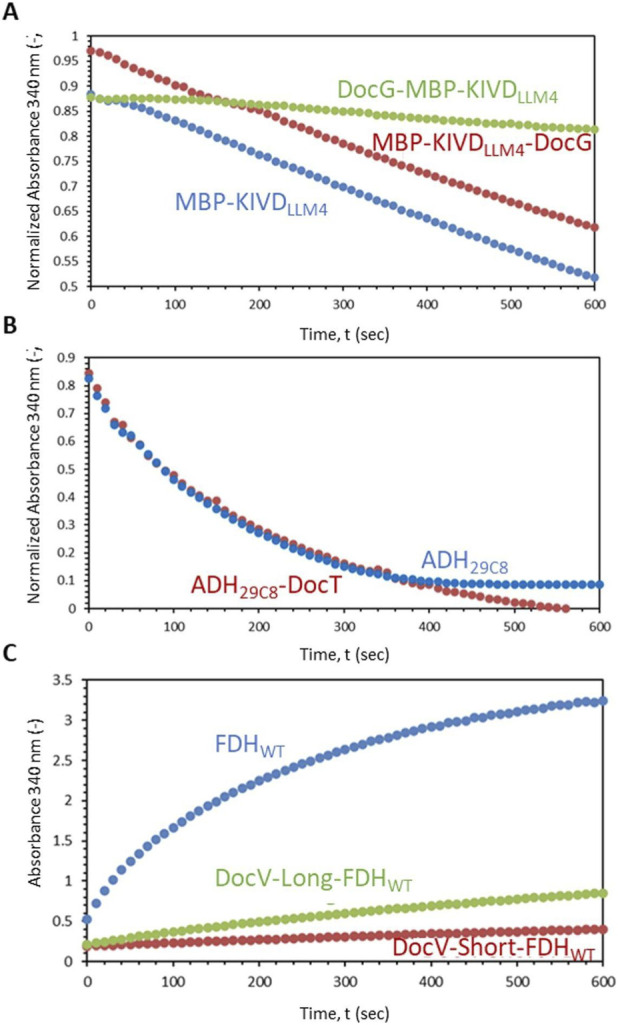
Enzyme activity assays. **(A)** Comparison of fused and non-fused MBP-KIVD_LLM4_ variants. The location of DocG in the labels in the figure denote its position in the respective fusion protein. Absorbance was normalized by dividing each value by the initial KIV absorbance. **(B)** Comparison of fused and non-fused ADH_29C8_ variants. Absorbance was normalized by initial NADH absorbance. **(C)** Comparison of fused and non-fused FDH_WT_, with the long- and short-linker variants.

Using the standard BL21 strain for KIVD expression and BL21 (DE3) pLysS for the expression of ADH and FDH, the enzymatic activities of the fusion constructs were compared to their corresponding wild-type enzymes (Figure 4). The N-terminal DocG fusion to MBP-KIVD_LLM4_ exhibited minimal activity (Figure 4A), which could be attributed to the reduced expression of the fusion protein. In comparison, the C-terminal fusion retained comparable activity to the wild-type protein, with activity values of 0.011 and 0.013 abs*sec^-1^*mg^-1^, respectively. This observation was consistent with ADH, where the fusion and native enzyme exhibited activities of 5.5 and 7.5 abs*sec^-1^*mg^-1^, respectively.

For FDH, the initial N-terminal DocV-FDH_WT_ fusion variant was active; however, it was discovered that DocV-FDH_WT_ with an (EAAAK)_3_ linker exhibited poor binding to the cohesin-bearing scaffoldin, possibly due to cleavage. Therefore, the (EAAAK)_3_ linker was replaced with alternative sequences, previously shown to support N-terminal dockerin fusions: (i) a 22-residue native linker (GGPGGPGTPSPDPGTQPGTGTP, “Long”) derived from a cellulosomal cellulase and (ii) a truncated nine residue variant (GTQPGTGTP, “Short”) (Caspi et al., 2009). Despite prior work showing excellent stability (Wong et al., 2019), FDH_WT_ exhibited reduced activity upon fusion to the dockerin for both linker variants (Figure 4C). The short-linker construct (DocV-Short-FDH_WT_) was approximately 4-fold less active than the long-linker variant. Since FDH is the cofactor-recycling enzyme and prior observations that lower overall FDH activity does not significantly impact the overall process (Wong et al., 2019), it was decided to proceed with the more active fusion-linker variant without further optimization.

### 4.3 Fusion protein binding to cellulose-immobilized scaffoldin

**FIGURE 5:**
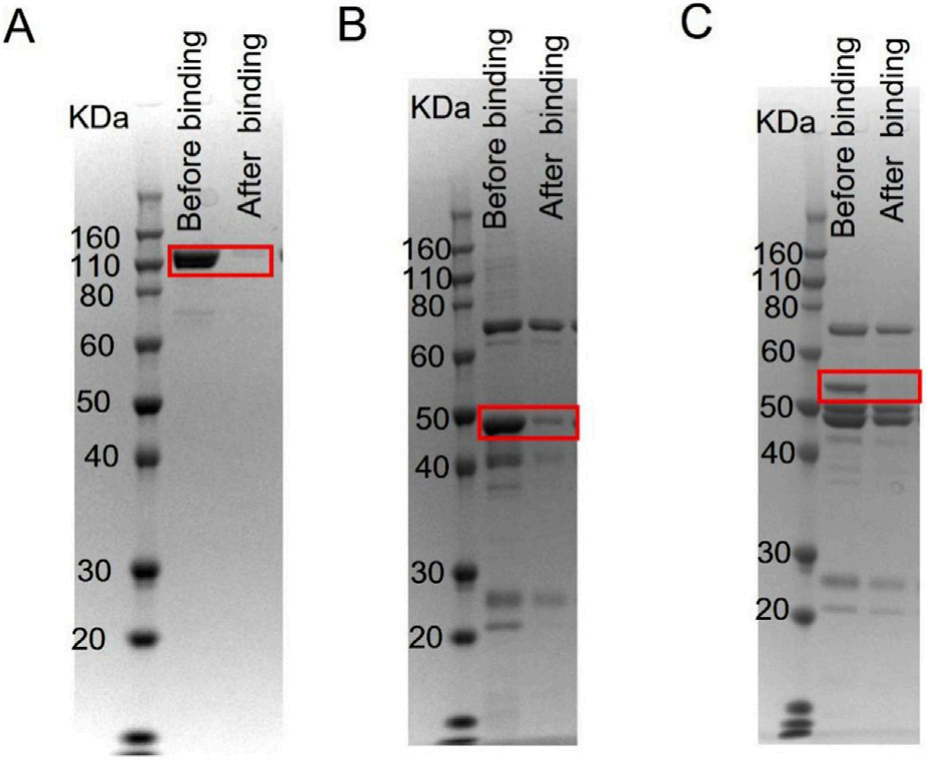
SDS-PAGE of enzyme binding to scaffoldin ScafGTV. **(A)** MBP-KIVD_LLM4_-DocG binding to the immobilized scaffoldin. **(B)** ADH_29C8_-DocT binding to ScafGTV. **(C)** DocV-Long-FDH_WT_ binding to ScafGTV. **(A–C)** “Before binding” denotes enzyme solution before interaction with the scaffoldin-bound cellulose matrix. “After binding” denotes the retentate retrieved after overnight interaction of the enzyme with the matrix. MBP-KIVD_LLM4_-DocG is 115 kDa and ADH_29C8_ DocT is 45 kDa, DocV-Long-FDH_WT_ is 53 kDa.

Figure 5 shows the initial binding results of the dockerin-containing fusion proteins to the trivalent cohesin-containing scaffoldin (ScafGTV). On an individual basis, all three enzymes bound to the scaffoldin as shown qualitatively by the disappearance of the corresponding dockerin-containing band in the retentate (i.e., after binding). The immobilized scaffoldin proved to be a tenacious binder of the three enzymes, capable of sequestering the provided enzyme almost completely. Ultimately, these results confirmed that the active fusion proteins, described above, could be successfully bound on an individual basis to the cohesin-containing scaffold.

**FIGURE 6:**
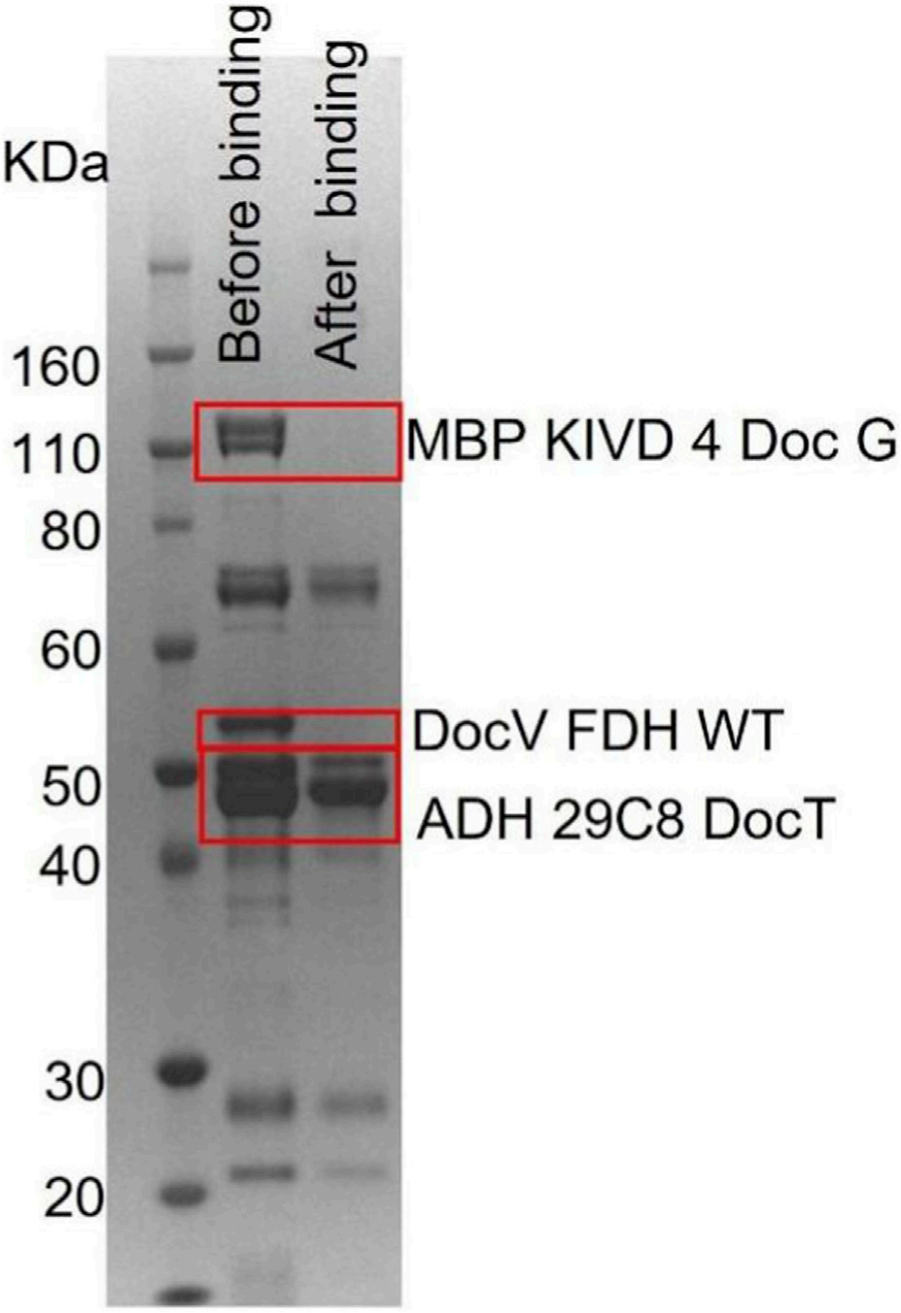
SDS-PAGE showing enzyme incorporation into scaffoldin ScafGTV. “Before” denotes enzyme solution before interaction with the scaffoldin-bound cellulose matrix. “After” denotes the retentate retrieved after overnight interaction of the enzymes with the matrix. MBP-KIVD_LLM4_-DocG is 115 kDa, ADH_29C8_-DocT is 45 kDa, and DocV-Long-FDH_WT_ is 53 kDa.

Beyond displaying the successful immobilization of each individual enzyme, an advantage of the scaffold system is that all three pathway enzymes bearing their respective dockerins can be localized on the same support. Confirmation of this trivalent binding is depicted in Figure 6. Analysis of the gel showed the existence of the expected bands for MBP-KIVD_LLM4_-DocG (115 kDa), ADH_29C8_-DocT (45 kDa), and DocV-Long-FDH_WT_ (52.8 kDa) prior to exposure to ScafGTV. Following mixture with ScafGTV, the gel showed a significant reduction in the bands corresponding to the KIVD and FDH fusions, indicating that these enzymes were bound to the scaffoldin. However, the ADH fusion band showed only a moderate reduction. This phenomenon is hypothesized to be due to two effects. First, the impurities corresponding to the FDH variant (as seen in Figure 5), with a similar molecular weight to ADH, could mask an apparent reduction of the gel band. Second, given the predicted location of ADH in the middle of the scaffoldin, steric crowding effects by the outer enzymes (KIVD and FDH) could contribute to reduced immobilization efficiency (Kokolaki et al., 2020). As ADH catalyzes the final step of the isobutanol pathway, incomplete incorporation of dockerin-fused ADH could influence overall pathway efficiency. Therefore, future work should include additional analysis of ADH immobilization efficiency and optimization strategies, including control of scaffoldin linker length, enzyme immobilization order and orientation, and variation of ADH concentration during immobilization. For example, immobilizing ADH first could prevent steric hindrance caused by adjacent enzyme molecules. In addition, sequential enzyme immobilization upon the scaffold could be used to facilitate characterization of individual enzymatic loading through gel densitometric analysis or orthogonal protein quantification methods. These studies would help to investigate the relationship between ADH incorporation, enzyme presentation, and overall pathway productivity.

### 4.4 Isobutanol production using scaffoldin based enzymatic pathway

Consistent with the previously reported epoxy-resin-based immobilized reaction system (Wong et al., 2019), the isobutanol production of the scaffoldin immobilized enzyme system was examined (Table 1). A relatively high initial concentration of KIV (102 mM) was chosen to due to the potential for greater productivity that could overcome the issue of ADH sensitivity isobutanol concentration. Under this elevated starting condition, preliminary results showed that both yield and titer were improved relative to the previous system. More specifically, the enhanced final concentration of isobutanol after 24 h was 80 mM (5.92 g/L), representing an overall yield of 78.4%.

**TABLE 1:** Comparison of epoxy resin immobilization^†^ to targeted scaffoldin immobilization.

Immobilization method	Initial ketoisovaleric acid, (mM)	Final ketoisovaleric acid, (mM)	Isobutanol, (mM)
Epoxy resin	64	0.95	27.6
Scaffoldin^∝^	102	0^‡^	80

^†^ Data adapted from Wong et al. (2019).

^‡^ Not detected or below limits of detection.

^∝^ Preliminary result (n = 1).

Although the initial KIV concentration differed between the two studies, this difference alone is unlikely to explain the improved performance. For example, in the previous epoxy-based system, increasing the initial KIV concentration from 2 mM to 64 mM increased the final isobutanol concentration but substantially reduced yield from approximately 95%–43%. This trend was attributed to the inhibition of ADH at elevated isobutanol concentrations (Wong et al., 2019). Therefore, further increasing the initial KIV concentration in the previous system to match the 102 mM used here would be expected to moderately increase the isobutanol titer, while likely further decreasing the overall yield. Alternatively, because the scaffoldin-based system achieved a 78.4% yield even at 102 mM KIV, it is reasonable to expect that operation at lower initial KIV concentration, such as 64 mM, would reduce final titer but could still exhibit improved yield relative to the epoxy-resin-based system.

While yield and titer are useful first-order performance indicators, they provide limited insight into the underlying process efficiency gains enabled by the scaffoldin based system. Therefore, to assess these improvements, it is necessary to define productivity metrics that account for reactor footprint, time, and enzyme usage (see Equations 1, 2). Using this framework, it becomes clear that the scaffoldin-based strategy contributed to approximately a 3-fold increase in volumetric productivity and an order-of-magnitude increase in enzymatic productivity relative to the epoxy-based system (Table 2). It is important to note, however, that the estimation of enzymatic productivity assumed a complete enzyme immobilization in both systems. While this assumption provided a consistent basis for comparison, the gel results shown in Figures 5, 6 suggested that immobilization efficiency may be incomplete for ADH. Therefore, the reported enzymatic productivity values should be interpreted as a lower bound estimate. This is because if the actual amount of immobilized enzyme was lower than the amount used in the estimation, then the true enzymatic productivity, defined relative to enzyme mass, would be higher.

**TABLE 2:** Comparison of process productivity for epoxy resin^†^ and scaffoldin immobilized enzymatic systems.

Immobilization method	Mass of enzyme utilized, (mg)	Reaction time, (h)	Titer, (mg/mL)	Productivity, P_v_ (mg mL^-1^ h^-1^)	Productivity, P_e_ (mL^-1^ h^-1^)
Epoxy resin†	2.7^‡^ ^,^ ^§^	24	2.1	0.09	0.03
Scaffoldin^∝^	0.7^‡^ ^,^ ^#^	24	5.9	0.25	0.34

^†^ Data adapted from Wong et al. (2019).

^∝^ Preliminary result (n = 1).

^‡^ Assumes complete immobilization of added enzyme.

^§^ Based on the following enzyme concentrations per mg of resin: ADH (0.1 mg/mg), FDH (1.99 mg/mg), and KIVD (0.6 mg/mg). Total resin = 1 mg.

^#^ Based on the following enzyme concentrations added to immobilization reaction (0.75 mL ea.): ADH (0.26 mg/mL), FDH (0.185 mg/mL), and KIVD (0.51 mg/mL).

The cohesion-dockerin complex was previously explored as a strategy for enzyme organization to promote substrate channeling (Rollin et al., 2013), including applications in yeast display production of methanol (Liu et al., 2013) as well as cell-free production of fructose 6-phosphate (You and Zhang, 2013), 1,3-propanediol (Xu et al., 2021), and xylonic acid (Lee et al., 2017). Following these previous systems, substrate channeling provides a plausible explanation for the enhanced productivity as it involves the funneling of an intermediate produced by one enzyme to another without the disadvantages of long-distance diffusion (Dueber et al., 2009). In terms of the isobutanol pathway, utilization of the scaffoldin-based system may increase the local concentration of the aldehyde intermediate near ADH, thereby driving the conversion to isobutanol forward, while also overcoming the diffusion constraints for the intermediate (Wheeldon et al., 2016).

Although substrate channeling was the intended design principle of this system, alternative factors could also have contributed to the observed increase in productivity. For example, the scaffoldin-based and epoxy-resin-based systems differ in resin architecture, porous structure, surface chemistry, and enzyme attachment protocol. These variables may influence enzyme loading, coupling efficiency, enzyme accessibility, immobilized enzyme stability, and enzyme activity. In addition, the larger molecular footprint and presentation of the scaffoldin complex could alter the amount and distribution of enzyme immobilized on the support, while also affecting accessibility to reactants and intermediates. Finally, differences in the porous structure may influence mass transport efficiency through the matrix. Therefore, while the improved productivity is consistent with enhanced substrate channeling, the comparison should be recognized as the combined effect of enzymatic organization, immobilization chemistry, ligand properties, and support architecture.

Future work should directly isolate the contribution of substrate channeling by comparing scaffoldin-mediated immobilization with appropriate non-co-immobilized and randomly co-immobilized controls using the same support material, surface chemistry, enzyme loading, and reaction conditions. Furthermore, comparison with isobutanol production using the same enzyme constructs in solution would help decouple the effects of enzyme engineering from substrate immobilization. Additional studies measuring pathway kinetics and enzyme activity would further clarify the extent to which enzyme colocalization contributes to observed productivity increases. This analysis could be further enhanced by comparing the mass transport properties of reactants and intermediates to observed enzyme pathway kinetics, using a dimensionless criterion known as the Damköhler number. Altogether, these studies would not only elucidate the extent of substrate channeling but also define design criteria for optimal enzyme engineering and support design.

Nevertheless, recent developments in cell-free systems have altered the field radically. A full glucose-to-isobutanol system has been developed, producing isobutanol titers up to 275 g/L, far exceeding that of even the best *E. coli* production schemes and ethanol fermentation in general (Sherkhanov et al., 2020). However, this impressive titer was accompanied by substantial limitations from a process-feasibility standpoint. The system required high enzyme loadings (approximately 5 g/L in a 15-mL aqueous phase) with addition of supplementary enzymes to sustain production.

Given these limitations, it is necessary to look beyond the attractive titer and evaluate performance from a process-feasibility standpoint using the framework described above. As shown in Table 3, the optimized full pathway system exhibited a 46-fold higher titer and an order of magnitude increase in volumetric productivity relative to the scaffoldin-based system. However, once enzyme usage is accounted for, the scaffoldin-based system exhibited roughly a 2.1-fold higher enzymatic productivity, suggesting the advantages of solid-phase scaffoldin-mediated immobilization. It is important to note that the enzyme basis used for the non-immobilized system includes the amount of enzyme required for the final two pathway steps (KivD-S and EcYahK) and the NADPH cofactor regeneration enzyme (TkGapN′), ensuring that the comparison is made on an equivalent and conservative basis. In addition to decreased enzymatic productivity, the glucose-to-isobutanol system relied on a liquid-liquid extraction system using an organic overlay with phenetole. While this approach was effective in driving isobutanol formation by shifting reaction equilibrium, it introduces a costly organic phase and further necessitates an energy intensive thermal downstream separation.

**TABLE 3:** Comparison of process productivity for immobilized and non-immobilized^‡^ enzymatic systems.

System type	Mass of enzyme utilized, (mg)	Reaction time, (h)	Titer, (mg/mL)	Productivity, P_v_ (mg mL^-1^ h^-1^)	Productivity, P_e_ (mL^-1^ h^-1^)
Scaffoldin^†^ (*Solid Phase*)	0.7^∝^ ^,^ ^‡^	24	5.9	0.25	0.34
No immobilization^§^ (*Aqueous Phase with organic overlay and bolus enzyme addition*)	15.5^#^	108	275	2.6	0.16

^†^ Preliminary result (n = 1).

^∝^ Assumes complete immobilization of added enzyme.

^‡^ Based on the following enzymatic concentrations added to immobilization reaction (0.75 mL ea.): ADH (0.26 mg/mL), FDH (0.185 mg/mL), and KIVD (0.51 mg/mL).

^§^ Data adapted from Sherkhanov et al. (2020). See Figure 5D and Supplementary Table S6.

^#^ Based on final 2 enzymes of metabolic pathway + NADPH, cofactor regeneration enzyme: KivD-S (0.15 mg/mL + second bolus), EcYahK (0.27 mg/mL), and TkGapN’ (0.46 mg/mL), respectively. Reactor volume = 15 mL. note, as the amount of KivD-S, within the second bolus was unclear, for estimation purposes, the second bolus of KivD-S, is assumed to double the enzymes concentration within the bioreactor (0.3 mg/mL total).

In contrast, the immobilized scaffoldin-based scheme described enables a potential flow-through system to control the product isobutanol concentration and is further compatible with low-energy separation strategies, such as membrane-based filtration. This flexibility and compatibility enable control of isobutanol concentration to ultimately favor the forward reaction. However, the optimized parameters and exceptional titers achieved by the full metabolic pathway cannot be ignored. Thus, an attractive solution would be to combine the systems, either by utilizing only the end pathway scaffold described or converting the full enzymatic pathway to a scaffoldin based system. To achieve this integration, further development is necessary given that the complete pathway involves 16 enzymes and the current scaffold incorporates only a limited number of cohesins (Sherkhanov et al., 2020). In this context, chimeric scaffoldins containing six divergent cohesins have been described (Moraïs et al., 2012), and a functional adapter scaffoldin system has been described which incorporated up to eight different enzymes (Stern et al., 2016). Theoretically, the number of cohesins can potentially be extended further to include all enzymes of the pathway, using the adaptor scaffoldin approach.

Finally, the glucose-to-isobutanol system demonstrated maintained isobutanol production over 108 h, while the solid-phase scaffoldin system was evaluated over a 24 h reaction time. Prolonged enzyme activity and sustained productivity are critical considerations for full-scale implementation. Therefore, future work should investigate not only the effect of extended reaction time on system performance but also repeated usage of the system on enzymatic stability, production rate, and overall process yield.

Ultimately, the scaffoldin-based reaction system (Bayer et al., 1994) presented here for the production of isobutanol represents a proof-of-concept platform that can be further optimized for a broad range of applications. The modularity of this system suggests substantial potential for application to alternative enzymatic pathways, as dockerins can be fused to diverse enzymes with minimal additional engineering. The preliminary results presented here represent a promising step forward in cell-free chemical production in comparison to previously reported systems; however, future work should evaluate the reproducibility and robustness of the described results. This is particularly important for cell free production systems, where batch-to-batch variability in expression machinery (*e.g.*, expression yield, enzyme purity, immobilization efficiency) can influence process performance. Therefore, the effects of this variation should be evaluated, especially prior to broader technology translation.

## 5 Conclusions

In this study, we present a proof-of-concept platform for the production of isobutanol by incorporating the enzymes of the KIV pathway onto an efficient trivalent cohesin-bearing scaffold system. This platform was designed to address limitations associated with previously reported epoxy-resin-mediated immobilization, in which pathway enzymes are irreversibly and randomly immobilized. Using this system, multiple dockerin-containing enzymes were optimized in terms of dockerin fusion location, linker length/composition, and expression conditions. The selected enzymes were then incorporated onto a complementary scaffoldin without disruption of selected protein functions.

This approach has deepened our understanding of the factors that govern efficient biofuel production. Preliminary results showed that the fully immobilized reaction produced a titer of 5.92 g/L and a 78.4% yield with higher volumetric (0.25 mg mL^-1^ h^-1^) and enzymatic productivity (0.34 mL^-1^ h^-1^) when compared to the previously reported epoxy-immobilized system. More broadly, comparison of these performance parameters shows that *in vitro* process evaluation should not rely on product titer alone but should consider enzyme usage and productivity per unit enzyme mass. This analysis is especially important for cell-free manufacturing, where enzyme cost and efficiency are major drivers of process feasibility.

Overall, this work represents an encouraging starting point for further improvement of the system by additional molecular engineering and tuning. Moreover, this approach offers an alternative way to design and expand the enzyme system to incorporate an extended isobutanol pathway, perhaps starting at glucose, or exploring other pathways for industrial chemical production.

## Data Availability

The original contributions presented in the study are included in the article/Supplementary Material, further inquiries can be directed to the corresponding authors.

## References

[B1] Artzi L. Bayer E. A. Moraïs S. (2017). Cellulosomes: bacterial nanomachines for dismantling plant polysaccharides. Nat. Rev. Microbiol. 15 (2), 83–95. 10.1038/nrmicro.2016.164 27941816

[B2] Bayer E. A. Morag E. Lamed R. (1994). The cellulosome--a treasure-trove for biotechnology. Trends Biotechnol. 12 (9), 379–386. 10.1016/0167-7799(94)90039-6 7765191

[B3] Bayer E. A. Belaich J.-P. Shoham Y. Lamed R. (2004). The cellulosomes: multienzyme machines for degradation of plant cell wall polysaccharides. Annu. Rev. Microbiol. 58, 521–554. 10.1146/annurev.micro.57.030502.091022 15487947

[B4] Callejon S. Sendra R. Ferrer S. Pardo I. (2017). Recombinant laccase from Pediococcus acidilactici CECT 5930 with ability to degrade tyramine. PloS One 12 (10), e0186019. 10.1371/journal.pone.0186019 29020076 PMC5636118

[B5] Caspi J. Barak Y. Haimovitz R. Irwin D. Lamed R. Wilson D. B. (2009). Effect of linker length and dockerin position on conversion of a Thermobifida fusca endoglucanase to the cellulosomal mode. Appl. Environmental Microbiology 75 (23), 7335–7342. 10.1128/AEM.01241-09 19820154 PMC2786427

[B6] Chen C.-T. Liao J. C. (2016). Frontiers in microbial 1-butanol and isobutanol production. FEMS Microbiology Letters 363 (5), fnw020. 10.1093/femsle/fnw020 26832641

[B7] Chen X. Zaro J. L. Shen W.-C. (2013). Fusion protein linkers: property, design and functionality. Adv. Drug Delivery Reviews 65 (10), 1357–1369. 10.1016/j.addr.2012.09.039 23026637 PMC3726540

[B8] Clark E. D. B. (2001). Protein refolding for industrial processes. Curr. Opinion Biotechnology 12 (2), 202–207. 10.1016/s0958-1669(00)00200-7 11287238

[B9] Dueber J. E. Wu G. C. Malmirchegini G. R. Moon T. S. Petzold C. J. Ullal A. V. (2009). Synthetic protein scaffolds provide modular control over metabolic flux. Nat. Biotechnology 27 (8), 753–759. 10.1038/nbt.1557 19648908

[B10] Dunlop M. J. (2011). Engineering microbes for tolerance to next-generation biofuels. Biotechnol. Biofuels 4 (1), 1–9. 10.1186/1754-6834-4-32 21936941 PMC3189103

[B11] Francis D. M. Page R. (2010). Strategies to optimize protein expression in *E. coli* . Curr. Protoc. Protein Sci. 61 (1), 5.24.21–25.24.29. 10.1002/0471140864.ps0524s61 20814932 PMC7162232

[B12] Friguet B. Chaffotte A. F. Djavadi-Ohaniance L. Goldberg M. E. (1985). Measurements of the true affinity constant in solution of antigen-antibody complexes by enzyme-linked immunosorbent assay. J. Immunological Methods 77 (2), 305–319. 10.1016/0022-1759(85)90044-4 3981007

[B13] Grimaldi J. Collins C. Belfort G. (2014). Toward cell‐free biofuel production: stable immobilization of oligomeric enzymes. Biotechnol. Progress 30 (2), 324–331. 10.1002/btpr.1876 24449684

[B14] Grimaldi J. Collins C. H. Belfort G. (2016). Towards cell‐free isobutanol production: development of a novel immobilized enzyme system. Biotechnol. Prog. 32 (1), 66–73. 10.1002/btpr.2197 26560680

[B15] Gupta M. Wong M. Jawed K. Gedeon K. Barrett H. Bassalo M. (2022). Isobutanol production by combined *in vivo* and *in vitro* metabolic engineering. Metab. Eng. Commun. 15, e00210. 10.1016/j.mec.2022.e00210 36325486 PMC9619177

[B16] Kokolaki M. L. Fauquier A. Renner M. (2020). Molecular crowding and diffusion-capture in synapses. iScience 23 (8), 101382. 10.1016/j.isci.2020.101382 32739837 PMC7399191

[B17] Lee C. C. Kibblewhite R. E. Paavola C. D. Orts W. J. Wagschal K. (2017). Production of D-xylonic acid from hemicellulose using artificial enzyme complexes. J. Microbiol. Biotechnol. 27 (1), 77–83. 10.4014/jmb.1606.06041 27666987

[B18] Lim S. Jung G. A. Glover D. J. Clark D. S. (2019). Enhanced enzyme activity through scaffolding on customizable self‐assembling protein filaments. Small 15 (20), 1805558. 10.1002/smll.201805558 30920729

[B19] Liu F. Banta S. Chen W. (2013). Functional assembly of a multi-enzyme methanol oxidation cascade on a surface-displayed trifunctional scaffold for enhanced NADH production. Chem. Commun. 49 (36), 3766–3768. 10.1039/c3cc40454d 23535691

[B20] Moraïs S. Morag E. Barak Y. Goldman D. Hadar Y. Lamed R. (2012). Deconstruction of lignocellulose into soluble sugars by native and designer cellulosomes. MBio 3 (6), e00508–e00512. 10.1128/mBio.00508-12 23232718 PMC3520109

[B21] Moraïs S. David Y. B. Bensoussan L. Duncan S. H. Koropatkin N. M. Martens E. C. (2016a). Enzymatic profiling of cellulosomal enzymes from the human gut bacterium, R uminococcus champanellensis, reveals a fine‐tuned system for cohesin-dockerin recognition. Environ. Microbiol. 18 (2), 542–556. 10.1111/1462-2920.13047 26347002

[B22] Morais S. Stern J. Kahn A. Galanopoulou A. P. Yoav S. Shamshoum M. (2016b). Enhancement of cellulosome-mediated deconstruction of cellulose by improving enzyme thermostability. Biotechnol. Biofuels 9 (1), 164. 10.1186/s13068-016-0577-z 27493686 PMC4973527

[B23] Pagès S. Bélaïch A. Bélaïch J. P. Morag E. Lamed R. Shoham Y. (1997). Species‐specificity of the cohesin‐dockerin interaction between Clostridium thermocellum and clostridium cellulolyticum: prediction of specificity determinants of the dockerin domain. Proteins Struct. Funct. Bioinforma. 29 (4), 517–527. 10.1002/(SICI)1097-0134(199712)29:4<517::AID-PROT11>3.0.CO;2-P 9408948

[B24] Rollin J. A. Tam T. K. Zhang Y.-H. P. (2013). New biotechnology paradigm: cell-free biosystems for biomanufacturing. Green Chem. 15 (7), 1708–1719. 10.1039/C3GC40625C

[B25] Sherkhanov S. Korman T. P. Chan S. Faham S. Liu H. Sawaya M. R. (2020). Isobutanol production freed from biological limits using synthetic biochemistry. Nat. Commun. 11 (1), 1–10. 10.1038/s41467-020-18124-1 32855421 PMC7453195

[B26] Slutzki M. Barak Y. Reshef D. Schueler-Furman O. Lamed R. Bayer E. A. (2012). Indirect ELISA-based approach for comparative measurement of high-affinity cohesin–dockerin interactions. J. Mol. Recognit. 25 (11), 616–622. 10.1002/jmr.2178 23108621

[B27] Soh L. M. Mak W. S. Lin P. P. Mi L. Chen F.Y.-H. Damoiseaux R. (2017). Engineering a thermostable keto acid decarboxylase using directed evolution and computationally directed protein design. ACS Synthetic Biology 6 (4), 610–618. 10.1021/acssynbio.6b00240 28052191

[B28] Stern J. Moraïs S. Lamed R. Bayer E. A. (2016). Adaptor scaffoldins: an original strategy for extended designer cellulosomes, inspired from nature. MBio 7 (2), e00083. 10.1128/mBio.00083-16 27048796 PMC4959524

[B29] Su Y. Zhang W. Zhang A. Shao W. (2020). Biorefinery: the production of isobutanol from biomass feedstocks. Appl. Sci. 10 (22), 8222. 10.3390/app10228222

[B30] Weick E.-M. Zinder J. C. Lima C. D. (2020). “Strategies for generating RNA exosome complexes from recombinant expression hosts,” in The Eukaryotic RNA Exosome: Methods and Protocols. Editors LaCava J. Vaňáčová Š. (New York, NY: Springer), 417–425. 10.1007/978-1-4939-9822-7_20 PMC8565498 31768988

[B31] Wheeldon I. Minteer S. D. Banta S. Barton S. C. Atanassov P. Sigman M. (2016). Substrate channelling as an approach to cascade reactions. Nat. Chemistry 8 (4), 299–309. 10.1038/nchem.2459 27001725

[B32] Wong M. Zha J. Sorci M. Gasparis C. Belfort G. Koffas M. (2019). Cell-free production of isobutanol: a completely immobilized system. Bioresour. Technol. 294, 122104. 10.1016/j.biortech.2019.122104 31542497

[B33] Xu Q. Alahuhta M. Hewitt P. Sarai N. S. Wei H. Hengge N. N. (2021). Self-assembling metabolon enables the cell free conversion of glycerol to 1, 3-Propanediol. Front. Energy Res. 9 (NREL/JA-2700-80697), 680313. 10.3389/fenrg.2021.680313

[B34] Yaron S. Morag E. Bayer E. A. Lamed R. Shoham Y. (1995). Expression, purification and subunit-binding properties of cohesins 2 and 3 of the Clostridium thermocellum cellulosome. FEBS Letters 360 (2), 121–124. 10.1016/0014-5793(95)00074-j 7875315

[B35] You C. Zhang Y. H. P. (2013). Self-assembly of synthetic metabolons through synthetic protein scaffolds: one-step purification, Co-immobilization, and substrate channeling. ACS Synth. Biol. 2 (2), 102–110. 10.1021/sb300068g 23656373

