# Supplementary material for: Beyond enzyme engineering: ordered enzyme immobilization drives enhanced productivity in vitro
Source: Front Bioeng Biotechnol. 2026 Jul 29;14:1879695. doi: 10.3389/fbioe.2026.1879695 (PMC13461601; doi:10.3389/fbioe.2026.1879695)
Supplement: Supplementary file 1 [file Table1.docx]

## Wednesday, May 6, 2026

**Submission to *Frontiers in Bioengineering and Biotechnology***

**Supplementary Information for Main Manuscript entitled:**

**“Beyond Enzyme Engineering: Ordered enzyme immobilization drives enhanced productivity *in vitro*"**

**Authors:** Matthew Wong^1,†^, Thomas G. Neuman^1,†^, Md Anarul Hoque^1^, Sarah Moraïs^2^, Edward Bayer^2,3^, Georges Belfort^1,*^, Mattheos Koffas^1,*^,

**Affiliations:**

^1^Howard P. Isermann Department of Chemical and Biological Engineering and the Center for Biotechnology and Interdisciplinary Studies, Rensselaer Polytechnic Institute, Troy, NY 12180-3590, United States

^2^Faculty of Natural Sciences, Life Sciences, Ben-Gurion University of the Negev, Beer-Sheva 8410501, Israel.

^3^Department of Biomolecular Sciences, The Weizmann Institute of Science, Rehovot 7610001, Israel.

^†^These authors contributed equally to this work.

*Corresponding authors

(518)-424-5803 [belfog@rpi.edu](mailto:belfog@rpi.edu); ORCID iD: 0000-0002-7314-422X

(518)-253-5558 [koffam@rpi.edu](mailto:koffam@rpi.edu)

The following figures are of SDS-PAGE gels dealing with the expression of the dockerin-bearing fusion proteins. The tables detail primers for cloning and mutagenesis of the fusion proteins.

Figure S1: SDS-PAGE gel of N-terminal dockerin-containing fusion enzymes. F: flowthrough, W#: wash number, E: elution. The purified band is highlighted by the red box. Theoretical location of purified bands highlighted with green box. MBP-KIVD_LLM4_-DocG is 115 kDa, ADH_29C8_-DocT is 45 kDa, and FDH_WT_-DocV is 50 kDa.

Figure S2: SDS-PAGE gel of dockerin-bearing N-terminal ADH-DocT. L: lysate, LD: diluted lysate, F: flowthrough, W#: wash number, E: elution. The expressed band is highlighted by the red box. The estimated MW of ADH-DocT is 45 kDa

Figure S3: SDS-PAGE gel of DocV-FDH_WT_ containing short and long linkers. E: elution and C: buffer exchanged. Expressed band highlighted with red box. The expressed band is highlighted by the red box. The estimated MWs of DocV-Short-FDH_WT_ is 51.6 kDa and DocV-Long-FDH_WT_ is 52.8 kDa.

Table 1: Primers for assembly of fusions^a^

| Protein | Primer | Sequence | Melting Temp °C^a^ | GC% |
| --- | --- | --- | --- | --- |
| DocT-ADH | Forward | AAGAAGGAGATATACATATGCCAGAAAGCAGCAGC | 59 | 43 |
|  | Reverse | ACTACTGCTGCTTTCATATGGGCGCGCCATTTCGC | 61 | 54 |
| ADH-DocT | Forward | TATATACTCGAGCCAGAAAGCAGCAGCG | 56 | 50 |
|  | Reverse | CAAATTTCCTGTAGCAGAAAATCCTCTCGAG | 56 | 42 |
| DocG-MBP-KIVD | Forward | TGCCGCGCGGCAGCCATATGCCAGAAGAAGC | 61 | 65 |
|  | Reverse | CCTTCTTCGATTTTCATATGGATTTTGGCGCGCCATTTCG | 60 | 45 |
| MBP-KIVD-DocG | Forward | TTGCTGAACAAAATAAATCACCAGAAGAAGCAAACAAGGGAG | 59 | 38 |
|  | Reverse | TATTTGATGCCTCTAGATTACTCGAGCTTACCCAGTAAGCC | 59 | 44 |
| DocV-FDH | Forward | AAGAAGGAGATATACATATGTCTAGAGATCTGCGGTACCGTCTC | 50 | 43 |
|  | Reverse | ACTAAAACGATCTTCATATGGGCGCGCCATTTCGC | 61 | 49 |
| FDH-DocV | Forward | AACACGATAAGAAACTCGAGAGATCTGCGGTACCGTCTC | 59 | 49 |
|  | Reverse | GTGGTGGTGGTGCTCGAGTTGTTCTTCAACTGGGAATAAGGATATTTTTCC | 55 | 45 |

^a^ Purchased from Integrated DNA Technologies (Coralville, Iowa)

Table 2: Primers for DocV-Linker-FDH Design^a^

| Purpose | Primer | Sequence | Melting Temp °C^a^ | GC% |
| --- | --- | --- | --- | --- |
| Stop insertion | Forward | CACGATAAGAAACTCGAGTGACACCACCACC | 56 | 52 |
|  | Reverse | GGTGGTGGTGTCACTCGAGTTTCTTATCGTG | 56 | 52 |
| His insertion | Forward | CTTTAAGAAGGAGATATACATATGTCTAGACACCAC | 59 | 36 |
|  | Reverse | ATAAATTTATGAGACGGTACCGCCAG | 59 | 42 |
| Linker insertion | Forward | GTACTCGGAAAAATATCCTTATTCCCAGTTGAAG | 60 | 38 |
|  | Reverse | GCGTGTTTACCAGCATCATATAAGACTAAAACG | 61 | 39 |

^a^ Purchased from Integrated DNA Technologies (Coralville, Iowa)
